# Increased food intake with oxyntomodulin analogues

**DOI:** 10.1016/j.peptides.2015.09.006

**Published:** 2015-11

**Authors:** Samantha L. Price, James S. Minnion, Stephen R. Bloom

**Affiliations:** Department of Investigative Medicine, Imperial College, London W12 0NN, United Kingdom

**Keywords:** Gcgr, glucagon receptor, GLP‑1r, GLP‑1 receptor, Oxyntomodulin, Glucagon, GLP-1, cAMP

## Abstract

Oxyntomodulin analogues offer a novel treatment for obesity. However during analogue screening in a rat model increased food intake was consistently observed. To further investigate this finding, a series of representative analogues (OXM14 and OXM15) and their Glu-3 equivalents (OXM14E3 and OXM15E3) were administered to rats for 7 days and food intake and bodyweight measurements taken. To investigate the role of glucagon receptor activation glutamate (Glu/E) was substituted at amino acid position 3. GLP-1 and glucagon receptor efficacy of the oxyntomodulin analogues and their Glu-3 counterparts were measured at the rat receptors in vitro. Doses of 25 n mol/kg of OXM14 and OXM15 increased food intake by up to 20%. Bodyweight was not significantly increased. Food intake was not increased with the Glu-3 peptides, indicating that a glucagon receptor mechanism may be responsible for the increase in food intake.

## Highlights

• In rats OXM analogues decreased bodyweight without decreasing food intake.
• Food intake was often increased at low OXM doses.
• Comparison with Glu-3 substituted GLP-1 receptor selective analogues.
• No increase in food intake seen with Glu-3 peptides.
• Suggests a glucagon receptor mechanism may be involved in increasing food intake.

## 1 Introduction

Oxyntomodulin is the 29 amino acids of glucagon with an additional C-terminal octapeptide tail. It is a product of the proglucagon precursor peptide along with glucagon-like peptide-1 (GLP-1), glucagon and glucagon-like peptide-2 (GLP-2) and is co-secreted post prandially with GLP-1 from L-cells in the distal intestine [21]. A specific oxyntomodulin receptor has not been identified; the peptides proposed mechanism of action is by activation of both the glucagon and GLP-1 receptors, although with reduced efficacy compared to the native ligands [22], [23], [32]. In vivo studies have shown that peripheral administration of oxyntomodulin causes weight loss in rodents [1], [5], [6], [25] and humans [4], [36], [37] and both glucagon and oxyntomodulin have been shown to increase energy expenditure in these species [7], [19], [34], [36], [37]. Based on findings in mice it has been suggested that oxyntomodulin reduces food intake via activity at the GLP-1 receptor, while its activity at the glucagon receptor causes weight loss by increasing energy expenditure [25]. Glucagon stimulates glycogenolysis and gluconeogenesis, elevating blood glucose, and administration of glucagon causes hyperglycaemia [3]. However, oxyntomodulin administration does not elevate blood glucose [36], [37]. Recent studies have suggested that chronic oxyntomodulin administration improves glucose tolerance by increasing insulin secretion via the GLP-1 receptor and additionally by reducing weight [12], [25].

Despite the beneficial effects of oxyntomodulin on weight and glucose homeostasis, the very short half-life of native oxyntomodulin means it is not used therapeutically [24]. The proglucagon peptides, for example, are susceptible to degradation by the enzyme dipetidylpeptidase-IV (DPPIV) [39]. Our group, and several others, have reported that oxyntomodulin can be successfully modified to produce long acting analogues with sustained food intake reduction and enhanced body weight loss [2], [11], [23], [26].

As part of a proprietary drug development program, analogues of oxyntomodulin were designed with modifications in regions susceptible to degradation. The changes made were conservative, thus the analogues were not derivatized, were of similar length to the native peptide, had no cross linkages and included only natural amino acids. All analogues were based on the 29 amino acids of glucagon and contained 5–7 single substitutions between residues 12–29, with 2 or 3 additional amino acid residues at the C-terminal. During screening it was observed that when administered to rats, the numerous analogues tested did cause significant reductions in bodyweight but, unexpectedly had either no effect on food intake, or actually caused an increase. The current paper focuses on a representative sample of these oxyntomodulin analogues to further investigate the mechanism behind the increase in food intake and to assess the impact these findings might have on the design and therapeutic use of oxyntomodulin analogues.

## 2 Materials and methods

### 2.1 Peptides

All oxyntomodulin analogues tested (OXM8, OXM9, OXM10, OXM14, OXM15, OXM14E3, OXM15E3) and native peptides (Exendin-4 and human glucagon, GLP-1_(7-36NH2)_ and oxyntomodulin) were custom synthesized by Insight Biotechnology Limited (Middlesex, UK) using solid phase peptide synthesis (SPPS) methodology and purified by reverse-phase preparative HPLC. Peptide purity was determined by reverse-phase HPLC and by Matrix-assisted laser desorption ionization mass spectroscopy (MALDIMS). All peptides had a purity of >90%.

### 2.2 In vitro cAMP accumulation

The effects of oxyntomodulin analogues and native GLP-1, glucagon and oxyntomodulin on cyclic adenosine monophosphate (cAMP) accumulation were assessed in Chinese hamster ovary (CHO-K1) cells over-expressing the rat glucagon receptor (cDNA from Origene, Maryland, USA) and Chinese hamster lung (CHL) cells over-expressing the rat GLP-1 receptor (a kind gift from Professor Bernard Thorens, University of Lausanne, Switzerland). For cAMP studies, cells were seeded at a density of 150000 cells/ml in a 48 well plate (Nunc, VWR International Inc., Chicago, USA) and incubated for 24 h. Cells were serum starved for 1 h and then incubated for 30 mins at room temperature with a range of peptide concentrations diluted in serum free DMEM (Sigma–Aldrich, Dorset, UK) in the presence of 1 mM IBMX (3-isobutyl-1-methylxanthine, Sigma–Aldrich). After incubation, medium was removed and cells lysed with 0.1 M HCl +0.5% Triton-X. cAMP levels were measured using an ELISA kit according to manufacturer’s instructions (ADI-900-066, Enzo Life Sciences, New York, USA), optical density was read at 405 nm on a Biotek ELx808 (Wolf Laboratories, York, UK). EC_50_’s were calculated using Prism v5 (GraphPad Software Inc., San Diego, CA, USA). Concentrations were applied in duplicate or triplicate per experiment and each experiment repeated a minimum of 3 times.

### 2.3 Animals

Male Wistar rats (Charles River, Margate, UK) were maintained in individual cages under controlled temperature (21–23 °C) and light conditions (12:12 light–dark cycle, lights on at 0700 h) with ad‑libitum access to food (RM1 diet; SDS, Witham, UK) and water unless otherwise stated. All animal procedures were approved under the British Home Office Animals (Scientific Procedures) Act 1986 (Project License 70/7236).

### 2.4 Seven day food intake and bodyweight studies

Rats were weighed at 09:00 on the morning of study commencement and randomisation to treatment groups was stratified by bodyweight. A subcutaneous injection of vehicle (diluent) or peptide (maximum volume 100 μl/kg, reconstituted in ZnCl_2_ slow release formulation) was administered at 1600 h daily and food and bodyweight measured at the time of administration. A minimum of 7 animals per group was required based on previous observations. n numbers varied between experiments, see figure legends for the exact sample size of each group.

### 2.5 Statistical analysis

All food and bodyweight data are presented as mean ± SEM. Data was normally distributed according to the D’Agostino & Pearson omnibus normality test or the Shaprio–Wilk normality test for groups where *n *= 7. Between group variances were similar according to Bartlett’s test for equal variances. Differences in 0–7 days cumulative food intake and bodyweight data were analysed using one way ANOVA and post hoc tests with Dunnett’s or Bonferroni corrections for multiple comparisons (Prism v5, GraphPad Software Inc. San Diego, CA, USA). cAMP EC_50_’s were calculated using a sigmoidal dose response curve and non-linear regression (Prism v5). EC_50_ data is presented as a ratio of analogue average EC_50_/average glucagon EC_50_ or GLP-1 EC_50_. Average EC_50_’s were calculated from a minimum of 3 separate experiments. In all cases *P* < 0.05 was considered statistically significant.

## 3 Results

### 3.1 Oxyntomodulin and analogues favour glucagon receptor activity at the rat receptors

At the rat receptors, oxyntomodulin and analogues showed greater efficacy at the glucagon receptor than the GLP1 receptor (Table 1). The analogues had greater activity than oxyntomodulin at both the rat glucagon and GLP-1 receptors. The Glu-3 peptides OXM14E3 and OXM15E3 had similar efficacy at the rat GLP-1 receptor compared to OXM14 and OXM15 respectively, however their efficacies at the rat glucagon receptor were 165 and 27 fold lower respectively (Table 1).

### 3.2 Oxyntomodulin analogues reduce bodyweight without reducing food intake

**Fig. 1:**
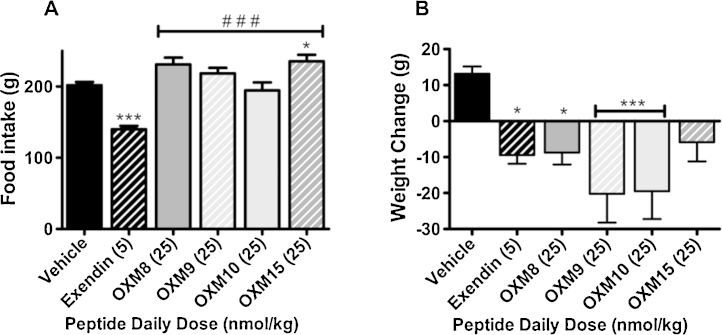
Daily S/C 25 n mol/kg oxyntomodulin analogue administration increased cumulative 7 day food intake (A) and reduced bodyweight (B) compared to vehicle administration. Average starting weight 506 g. Vehicle, OXM9, OXM10 *n *= 8/group; all other groups *n* = 7/group. Data represent mean ± SEM. Significance calculated using one way ANOVA (A, *F* = 16.78, *P* < 0.0001; B, *F* = 5.074, *P* = 0.0011) with Bonferroni post hoc test (*P < 0.05, ***P < 0.001 vs vehicle controls, ^###^*P* < 0.001 vs Exendin-4 group).

A daily dose of 25 n mol/kg OXM15 caused a significant 17% increase in cumulative food intake by the end of the 7 day treatment period compared to vehicle controls (*P* < 0.001; *n *= 7–8; Fig. 1A).

Daily administration of 25 n mol/kg OXM8, OXM9 and OXM10, significantly reduced 7 day bodyweight by 4–6% compared to vehicle controls (OXM8: *P* < 0.01, OXM9: *P* < 0.001, OXM10: *P* < 0.001; *n *= 7–8; Fig. 1B).

Seven day food intake was significantly increased in all groups administered daily 25 n mol/kg doses of OXM analogues compared to 5 n mol/kg Exendin-4 (*P* < 0.001; Fig. 1A).

### 3.3 Lower doses of OXM14 and OXM15 reduce weight gain without decreasing food intake

**Fig. 2:**
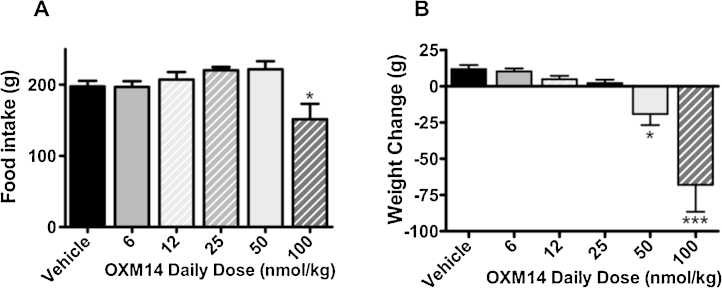
Cumulative 7 day food intake (A) and body weight change (B) in response to daily SC injections of various doses of OXM14 or vehicle. Average starting weight 577 g. Vehicle *n *= 9/group; all other groups *n* = 8/group. Data represent mean ± SEM. Significance calculated using one way ANOVA (A, *F* = 4.64, *P* = 0.0018; B, *F* = 13.58, *P* < 0.0001) with Dunnett’s post hoc test (*P < 0.05, ***P < 0.001 vs vehicle controls).

There was a dose dependent decrease in bodyweight during 7 day administration of OXM15 and similar analogue OXM14. There was trend towards a dose dependent increase in food intake with lower doses of both OXM14 and OXM15 compared to vehicle controls. Doses of 12-, 25- and 50 n mol/kg of OXM14 non significantly increased food intake by 5%, 11% and 12% respectively compared to vehicle controls (Fig. 2A). A daily 100 n mol/kg dose of OXM14 significantly decreased food intake by 23% compared to vehicle controls (*P* < 0.05, Fig. 2A). Doses of 12- and 25 n mol/kg decreased 7 day bodyweight non significantly by 1.2% and 1.7% respectively compared to vehicle controls. Doses of 50 n mol/kg and 100 n mol/kg of OXM14 significantly decreased bodyweight by 5% and 12% respectively compared to controls (*P* < 0.05, *P* < 0.001; *n *= 8–9; Fig. 2B).

**Fig. 3:**
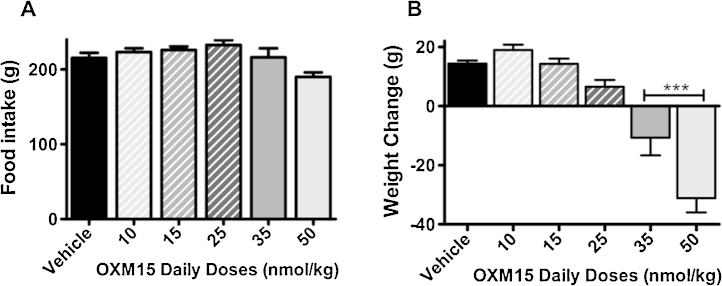
Cumulative 7 day food intake (A) and bodyweight change (B) after daily administration of OXM15 at various doses. Average starting weight 542 g. Vehicle, OXM(50), OXM(10) *n* = 9/group; all other groups *n* = 10/group. Data represent mean ± SEM. Significance calculated using one way ANOVA (A, *F* = 3.503, *P* = 0.0089; B, *F* = 27.13, *P* < 0.0001) with Dunnett’s post hoc test (***P < 0.001 vs vehicle controls).

Doses of 10-, 15- and 25 n mol/kg of OXM15 increased 7 day food intake by 4%, 5% and 8% respectively compared to vehicle controls. A daily 50 n mol/kg dose of OXM15 non significantly decreased food intake by 12% compared to controls (Fig. 3A). Seven day bodyweight was non significantly decreased by 1.4% by daily administration of 25 n mol/kg OXM15 (Fig. 3B). Daily doses of 35 n mol/kg and 50 n mol/kg OXM15 significantly reduced 7 day bodyweight by 5% and 8% respectively compared to controls *(P* < 0.001; *n *= 9–10; Fig. 3B).

### 3.4 No increased food intake with reduced glucagon receptor activity

To further investigate the effects of glucagon receptor activity on food intake, OXM14 and OXM15 were re-synthesized with the position 3 glutamine (Gln/Q) substituted for glutamate (Glu/E). This substitution had previously been reported to significantly diminish glucagon receptor activity without affecting GLP-1 receptor activity [12]. In-line with previous findings using Glu-3 peptides, the efficacy of OXM14E3 and OXM15E3 at the glucagon receptor were 173 and 27 times lower compared to OXM14 and OXM15 respectively, while their GLP1r activity was unaffected (Table 1).

**Fig. 4:**
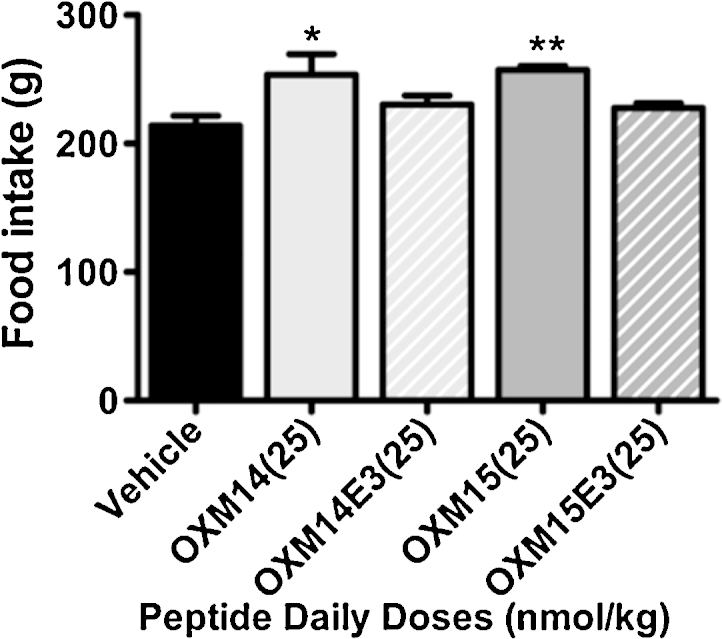
Cumulative 7 day food intake in response to daily 25 n mol/kg SC injections of OXM14, OXM14E3, OXM15, OXM15E3 or vehicle. Vehicle *n* = 8/group; all other groups *n* = 7. Data represent mean ± SEM. Significance calculated using one way ANOVA (*F* = 5.258, *P* = 0.0029) with Dunnett’s post hoc test (*P < 0.05, **P < 0.01 vs vehicle controls).

As seen in the previous experiments; repeated daily administration of 25 n mol/kg OXM15 significantly increased cumulative food intake over 7 days by 20% (*P* < 0.01), and OXM14 increased cumulative food intake by 19% (*P* < 0.05; *n *= 7; Fig. 4).

Neither OXM14E3 or OXM15E3 significantly increased cumulative food intake compared to vehicle controls (Fig. 4).

## 4 Discussion

This study suggests that analogues of oxyntomodulin can cause an increase in food intake in rats while generating significant reductions in bodyweight, and that the increased consumption may involve a glucagon receptor mediated mechanism. This finding seems to be a rat specific phenomenon which may be of interest in the development of oxyntomodulin analogues for therapeutic treatment of obesity in humans.

Previous studies have reported the use of oxyntomodulin analogues with a glutamate at amino acid position 3 as a tool to investigate the contribution of glucagon receptor activity to the effects on food intake and bodyweight. Thus, changing the position 3 glutamine of oxyntomodulin to glutamate (as found in GLP-1 and its natural analogue exendin-4) severely diminishes glucagon receptor activity without affecting the activity at the GLP-1 receptor, essentially producing a GLP-1 receptor selective analogue [12], [25], [31]. Comparing the effects of oxyntomodulin analogues with their Glu-3 counterparts in a chronic study therefore enables investigation of the contribution of glucagon receptor activity to oxyntomodulin function. In the current study, no increase in food intake was seen with GLP-1 selective Glu-3 analogues OXM14E3 and OXM15E3, suggesting that a glucagon receptor mechanism may be involved in mediating the increase in food intake. An increase in energy expenditure is seen following peripheral administration of glucagon in rodents [8] and humans [34]. Therefore in the current study the increase in food intake may be a compensatory response to a chronic increase in energy expenditure. A similar pattern of increased food intake without an increase in bodyweight is observed in patients with thyrotoxicosis and this is generally accepted to be a compensatory response to the hypermetabolic state induced by high levels of thyroid hormone [13]. A previous study has reported an acute hyperphagic response to IP glucagon administration in rats [18] and they attributed this to the low dose of 50 μg/kg (14 n mol/kg) that was used. Although glucagon has been suggested to acutely reduce food intake, this is typically observed following administration of higher doses [15], [17], [20], [30]. Also, several studies that have reported an acute reduction in food intake administered glucagon directly into the portal circulation [14], [16], [29], [35]. This could have produced different effects on food intake as it would have resulted in high glucagon levels local to the liver and could be considered less physiological than peripheral administration. No previous studies have investigated the effects of chronic exposure to glucagon on rodent food intake, probably due to the difficulties imposed by glucagon’s short half-life.

Previous studies using native oxyntomodulin and longer lasting dual analogues have reported a reduction in food intake after repeated peripheral administration in rodents [6], [9], [10], [23], [26], [31] and humans [36], [37]. The range of OXM14 and OXM15 doses that increase food intake are quite narrow and are generally lower than the doses of 50 n mol/kg [6], 1100 n mol/kg [25] and 1400 n mol/kg [23] of oxyntomodulin used in several prior chronic studies. Additionally, methods of peptide administration differ: some studies infused peptides [25] while others administered peptide every 2–4 days [9], [31] or twice daily [6], [28]. Liu et al. [26] did administer their dual analogue to rats at lower doses of 5 n mol/kg and 20 n mol/kg. However, their particular analogue had a much greater efficacy at the GLP-1 receptor, compared to the oxyntomodulin analogues in the current study, therefore any potential increase in food intake could have been masked by enhanced GLP-1 receptor activity causing a dominant reduction in food intake. Both native glucagon and oxyntomodulin have short half-lives and are also unstable in solution which makes it difficult to study the effects of chronic administration; Alzet pumps are problematic and frequent injections would involve significant handling and consequential animal stress.

Interestingly, the increase in food intake with low doses of oxyntomodulin analogues was not replicated in a mouse model (unpublished observations), indicating that this could be a species specific effect. A number of previous studies with oxyntomodulin analogues have been carried out using only mouse models [9], [23], [27], [28], [31], but there is no evidence as to whether human responses are more similar to rats or mice. These findings highlight the importance of testing in more than one model, particularly for dual analogues where differential efficacy may occur.

Oxyntomodulin activates both the human glucagon receptor and GLP-1 receptor with reduced efficacy compared to the native peptides [22], [23], [32]. There is a paucity of information in published literature comparing native oxyntomodulin activity at the rat glucagon receptor and rat GLP-1 receptor. Similar to previous findings at the human [23] and mouse [25] receptors, we observed that oxyntomodulin activity at the rat receptors favoured glucagon receptor activity. The oxyntomodulin analogues studied in the current paper also favoured glucagon receptor activity, but with greater efficacy at both rat receptors compared to native oxyntomodulin.

There was a trend towards an increase in food intake with low doses of both analogues up to a ‘threshold dose’, beyond which food intake was then decreased. It could be postulated that the threshold point may reflect the dose at which there is sufficient GLP1r activity to cause a reduction in food intake. There is a difference in threshold dose between OXM14 and OXM15 with a higher dose of OXM14 required to cause a reduction in food intake. It is likely this can be attributed to differences in their ratio of rat GLP-1 receptor to rat glucagon receptor activity. However, OXM14 has a greater efficacy at the rat GLP-1 receptor than OXM15 which would suggest a lower dose would be required to reduce food intake, thus highlighting the likelihood of a complex underlying interaction.

Exendin-4 is a naturally occurring GLP-1 receptor selective agonist with a longer half-life. The weight loss caused by administration of this peptide in standard chow fed animals is thought to be mainly due to reduction in food intake [33], [38]. In the initial experiment, all groups administered oxyntomodulin analogues lost similar amounts of weight as the exendin-4 group despite eating significantly more food. This indicates that the weight loss caused by the oxyntomodulin analogues may be due to increased energy expenditure as a result of glucagon receptor activity. With both OXM14 and OXM15 there was a trend towards a dose dependent reduction in bodyweight. At lower doses, food intake tended to increase as bodyweight decreased; indicating that below the ‘threshold point’ bodyweight loss is likely to be due to this proposed increased energy expenditure mediated via glucagon receptor activity. OXM15 has a similar ratio of activity at the rat glucagon receptor and rat GLP-1 receptor as OXM10 as well as a similar pharmacokinetic profile; however OXM15 significantly increased food intake despite generating less weight loss than OXM10. This suggests that the underlying receptor interactions involved with oxyntomodulin-like dual analogues, and probably oxyntomodulin, are complex and could include the degree of access to CNS appetite and energy expenditure controlling centres. Co-administration of glucagon and GLP-1 in rodents [30] and man [3] appears to produce synergistic effects on food intake, indicating probable interaction of the two peptides.

## 5 Conclusion

We have shown that in a rat model low doses of oxyntomodulin analogues often tend to increase food intake without increasing bodyweight.. Results with GLP-1 receptor selective Glu-3 analogues suggest that a glucagon receptor mediated mechanism may be involved in the increase in food intake. It is possible that the increase in food intake may be a compensatory response to an increase in energy expenditure mediated by glucagon receptor activity. It is currently unclear what impact these findings may have on the use of oxyntomodulin analogues in a clinical setting. Nausea limits the dose of GLP-1 analogues that can be used in man. It is unlikely that nausea is a feature of the oxyntomodulin analogues studied here, given the increase in food intake; however a conditioned taste aversion study in rats would help to clarify this.

## Figures and Tables

**Table 1:** cAMP EC_50_ ratios.

	EC_50_ ± SEM (nM)		EC_50_ ratio to native peptide (arbitrary units)	
	Rat Gcgr	Rat GLP-1r	Rat Gcgr	Rat GLP-1r
GLP-**1**	>1000	0.08 ± 0.01	ND	1.0
**Exendin-4**	>1000	0.06 ± 0.01	ND	0.75
**Glucagon**	0.34 ± 0.04	23.4 ± 5.88	1.0	297.2
**OXM**	11.4 ± 2.81	9.10 ± 0.40	33.1	115.7
**OXM8**	0.21 ± 0.05	2.18 ± 0.43	1.2	13.7
**OXM9**	0.48 ± 0.11	4.38 ± 0.81	0.6	27.7
**OXM10**	0.40 ± 0.10	1.08 ± 0.15	1.4	55.7
**OXM15**	0.38 ± 0.09	4.39 ± 1.17	1.1	55.8
**OXM15E3**	10.1 ± 1.66	6.99 ± 0.29	29.3	88.9
**OXM14**	0.23 ± 0.03	1.49 ± 0.25	0.7	19.0
**OXM14E3**	39.9 ± 2.61	2.18 ± 0.06	115.8	27.7

EC_50_ values for cAMP accumulation measured in CHO-K1 cells over expressing the rat glucagon receptor, or CHL cells over expressing the rat GLP-1 receptor after 30 min peptide stimulation. EC_50_’s for each peptide were calculated from the average EC_50_’s from 3–5 independent experiments. Receptor efficacy at the rat glucagon receptor is also expressed as a ratio of the EC_50_ of glucagon and of the EC_50_ of GLP1 at the rat GLP-1 receptor. A ratio value below 1 indicates enhanced efficacy, and above 1 decreased efficacy compared to the native peptide. ND—not determined, OXM—oxyntomodulin, Gcgr—glucagon receptor, GLP-1r—GLP-1 receptor.

## Glossary

Hyperglycaemia: High blood sugar
Hyperphagic: Increases food intake
Gluconeogenesis: A metabolic pathway resulting in the production of glucose from non-carbohydrate carbon substrates
Glycogenolysis: The breakdown of glycogen stores to produce glucose

## References

[1] Baggio L.L., Huang Q., Brown T.J., Drucker D.J. (2004). Oxyntomodulin and glucagon-like peptide-1 differentially regulate murine food intake and energy expenditure. Gastroenterology.

[2] Bianchi E., Carrington P.E., Ingallinella P., Finotto M., Santoprete A., Petrov A. (2013). A PEGylated analog of the gut hormone oxyntomodulin with long-lasting antihyperglycemic, insulinotropic and anorexigenic activity. Bioorg. Med. Chem..

[3] Cegla J., Troke R.C., Jones B., Tharakan G., Kenkre J., McCullough K.A. (2014). Coinfusion of low-dose GLP-1 and glucagon in man results in a reduction in food intake. Diabetes.

[4] Cohen M.A., Ellis S.M., Le Roux C.W., Batterham R.L., Park A., Patterson M. (2003). Oxyntomodulin suppresses appetite and reduces food intake in humans. J. Clin. Endocrinol. Metab..

[5] Dakin C.L., Gunn I., Small C.J., Edwards C.M., Hay D.L., Smith D.M. (2001). Oxyntomodulin inhibits food intake in the rat. Endocrinology.

[6] Dakin C.L., Small C.J., Batterham R.L., Neary N.M., Cohen M.A., Patterson M. (2004). Peripheral oxyntomodulin reduces food intake and body weight gain in rats. Endocrinology.

[7] Dakin C.L., Small C.J., Park A.J., Seth A., Ghatei M.A., Bloom S.R. (2002). Repeated I.C.V. administration of oxyntomodulin causes a greater reduction in body weight gain than in pair-fed rats. Am. J. Physiol. Endocrinol. Metab..

[8] Davidson I.W.F., Salter J.M., Best C.H. (1960). The effect of glucagon on the metabolic rate of rats. Am. J. Clin. Nutr..

[9] Day J.W., Gelfanov V., Smiley D., Carrington P.E., Eiermann G., Chicchi G. (2012). Optimization of co-agonism at GLP-1 and glucagon receptors to safely maximize weight reduction in DIO-rodents. Biopolymers.

[10] Day J.W., Ottaway N., Patterson J.T., Gelfanov V., Smiley D., Gidda J. (2009). A new glucagon and GLP-1 co-agonist eliminates obesity in rodents. Nat. Chem. Biol..

[11] Druce M.R., Minnion J.S., Field B.C., Patel S.R., Shillito J.C., Tilby M. (2009). Investigation of structure-activity relationships of oxyntomodulin (Oxm) using oxm analogs. Endocrinology.

[12] Du X., Kosinski J.R., Lao J., Shen X., Petrov A., Chicchi G.G. (2012). Differential effects of oxyntomodulin and GLP-1 on glucose metabolism. Am. J. Physiol. Endocrinol. Metab..

[13] Franklyn J.A., Boelaert K. (2012). Thyrotoxicosis. Lancet.

[14] Geary N., Le Sauter J., Noh U. (1993). Glucagon acts in the liver to control spontaneous meal size in rats. Am. J. Physiol..

[15] Geary N., Smith G.P. (1982). Pancreatic glucagon and postprandial satiety in the rat. Physiol. Behav..

[16] Geary N., Smith G.P. (1982). Pancreatic glucagon fails to inhibit sham feeding in the rat. Peptides.

[17] Geary N., Smith G.P. (1983). Selective hepatic vagotomy blocks pancreatic glucagon’s satiety effect. Physiol. Behav..

[18] Hell N.S., Timo-Iaria C. (1985). Increase of food intake induced by glucagon in the rat. Physiol. Behav..

[19] Heppner K.M., Habegger K.M., Day J., Pfluger P.T., Perez-Tilve D., Ward B. (2010). Glucagon regulation of energy metabolism. Physiol. Behav..

[20] Holloway S.A., Stevenson J.A. (1964). Effect of glucagon on food intake and weight gain in the young rat. Can. J. Physiol. Pharmacol..

[21] Holst J.J. (1997). Enteroglucagon. Annu. Rev. Physiol..

[22] Jorgensen R., Kubale V., Vrecl M., Schwartz T.W., Elling C.E. (2007). Oxyntomodulin differentially affects glucagon-like peptide-1 receptor beta-arrestin recruitment and signaling through galpha(s). J. Pharmacol. Exp. Ther..

[23] Kerr B.D., Flatt P.R., Gault V.A. (2010). (D-Ser2) Oxm[mPEG-PAL]: a novel chemically modified analogue of oxyntomodulin with antihyperglycaemic, insulinotropic and anorexigenic actions. Biochem. Pharmacol..

[24] Kervran A., Dubrasquet M., Blache P., Martinez J., Bataille D. (1990). Metabolic clearance rates of oxyntomodulin and glucagon in the rat: contribution of the kidney. Regul. Pept..

[25] Kosinski J.R., Hubert J., Carrington P.E., Chicchi G.G., Mu J., Miller C. (2012). The glucagon receptor is involved in mediating the body weight-lowering effects of oxyntomodulin. Obes. (Silver Spring).

[26] Liu Y.L., Ford H.E., Druce M.R., Minnion J.S., Field B.C., Shillito J.C. (2010). Subcutaneous oxyntomodulin analogue administration reduces body weight in lean and obese rodents. Int. J. Obes. (Lond.).

[27] Lynch A.M., Pathak N., Flatt Y.E., Gault V.A., O'Harte F.P., Irwin N. (2014). Comparison of stability, cellular, glucose-lowering and appetite supressing effects of oxyntomodulin analogues modified at the N-terminus. Eur. J. Pharmacol..

[28] Lynch A.M., Pathak N., Pathak V., O'Harte F.P., Flatt P.R., Irwin N. (2014). A novel DPP IV-resistant C-terminally extended glucagon analogue exhibits weight-lowering and diabetes-protective effects in high-fat-fed mice mediated through glucagon and GLP-1 receptor activation. Diabetologia.

[29] Martin J.R., Novin D. (1977). Decreased feeding in rats following hepatic-portal infusion of glucagon. Physiol. Behav..

[30] Parker J.A., McCullough K.A., Field B.C., Minnion J.S., Martin N.M., Ghatei M.A. (2013). Glucagon and GLP-1 inhibit food intake and increase c-fos expression in similar appetite regulating centres in the brainstem and amygdala. Int. J. Obes. (Lond.).

[31] Pocai A., Carrington P.E., Adams J.R., Wright M., Eiermann G., Zhu L. (2009). Glucagon-like peptide 1/glucagon receptor dual agonism reverses obesity in mice. Diabetes.

[32] Santoprete A., Capito E., Carrington P.E., Pocai A., Finotto M., Langella A. (2011). DPP-IV-resistant, long-acting oxyntomodulin derivatives. J. Pept. Sci..

[33] Szayna M., Doyle M., Betkey J.A., Holloway H.W., Spencer R.G.S., Greig N.H. (2000). Exendin-4 decelerates food intake, weight gain, and fat deposition in zucker rats. Endocrinology.

[34] Tan T.M., Field B.C., McCullough K.A., Troke R.C., Chambers E.S., Salem V. (2013). Coadministration of glucagon-like peptide-1 during glucagon infusion in humans results in increased energy expenditure and amelioration of hyperglycemia. Diabetes.

[35] Weick B.G., Ritter S. (1986). Dose-related suppression of feeding by intraportal glucagon infusion in the rat. Am. J. Physiol.- Regul. Integr. Comp. Physiol..

[36] Wynne K., Park A.J., Small C.J., Meeran K., Ghatei M.A., Frost G.S. (2006). Oxyntomodulin increases energy expenditure in addition to decreasing energy intake in overweight and obese humans: a randomised controlled trial. Int. J. Obes. (Lond.).

[37] Wynne K., Park A.J., Small C.J., Patterson M., Ellis S.M., Murphy K.G. (2005). Subcutaneous oxyntomodulin reduces body weight in overweight and obese subjects: a double-blind, randomized, controlled trial. Diabetes.

[38] Yang Y., Moghadam A.A., Cordner Z.A., Liang N.C., Moran T.H. (2014). Long term exendin-4 treatment reduces food intake and body weight and alters expression of brain homeostatic and reward markers. Endocrinology.

[39] Zhu L., Tamvakopoulos C., Xie D., Dragovic J., Shen X., Fenyk-Melody J.E. (2003). The role of dipeptidyl peptidase IV in the cleavage of glucagon family peptides: in vivo metabolism of pituitary adenylate cyclase activating polypeptide-(1-38). J. Biol. Chem..

